# Neurobiological and Behavioral Heterogeneity in Adolescents with Autism Spectrum Disorder

**DOI:** 10.3390/brainsci15101057

**Published:** 2025-09-28

**Authors:** Gerry Leisman, Rahela Alfasi, Robert Melillo

**Affiliations:** 1 Movement and Cognition Laboratory, Department of Physical Therapy, University of Haifa, Haifa 3498838, Israel; 2 Resonance Therapeutics Laboratory, University of the Medical Sciences of Havana, Havana 10400, Cuba; 3 Center for Developing Minds, Rockville Centre, New York, NY 11570, USA

**Keywords:** autism spectrum disorder (ASD), neurodevelopment, functional connectivity, executive function, social communication, behavioral regulation

## Abstract

Background: Adolescents with autism spectrum disorder (ASD) display distinct neurodevelopmental trajectories marked by atypical neural activation and white matter maturation compared to neurotypical peers. Introduction: While improvements in face recognition and cognitive skills occur during childhood and adolescence, individuals with ASD often experience a plateau in these areas as they transition to adulthood, impacting daily living, executive function, social cognition, and emotional awareness. Results: Neuroimaging studies reveal altered white matter growth and connectivity in brain regions associated with social processing, which may underlie these functional challenges. Intellectual disability further compounds developmental difficulties by limiting foundational abilities and slowing progress. Discussion: The multifaceted and persistent service needs spanning legal, educational, vocational, health, and psychosocial domains highlight the necessity for coordinated, individualized, and family-centered approaches, particularly during the transition to adulthood. Advances in research integrating genetic, neurobiological, and behavioral data hold potential for refining diagnostic subgroups and personalizing interventions. Conclusion: Continued advocacy and innovation in service delivery are essential to address gaps in adult support systems and enhance long-term outcomes for individuals with ASD.

## 1. Introduction

Autism spectrum disorder (ASD) is a neurodevelopmental condition characterized by persistent deficits in social communication and interaction, as well as restricted, repetitive patterns of behavior, interests, or activities. These symptoms typically emerge in early childhood and persist across the lifespan, though their manifestations may vary significantly over time and across individuals [1,2,3]. Adolescence, in particular, represents a period of rapid and often volatile neurobiological and psychosocial transformation, during which the developmental trajectory of individuals with ASD may diverge markedly from that of typically developing peers [4,5].

This divergence is compounded by the profound heterogeneity observed in both the neurobiological underpinnings and behavioral expressions of ASD. Neuroimaging studies in adolescents have revealed wide-ranging alterations in cortical thickness, white matter connectivity, and functional network dynamics that are not uniform across individuals with ASD [6,7,8]. Concurrently, behavioral profiles can vary from significant social withdrawal and sensory sensitivities to impulsivity, rigid thinking, and co-occurring conditions such as anxiety, ADHD, or mood disorders [9,10,11]. These complexities create substantial challenges for clinicians, educators, and families attempting to support adolescents with ASD during this critical developmental window.

While the literature has extensively addressed the core features of ASD and its early developmental presentation, there is comparatively less integration of findings specific to adolescence, especially with regard to the interplay between neurobiological changes and behavioral heterogeneity. The adolescent brain is undergoing synaptic pruning, myelination, and hormonal shifts that influence cognitive control, emotion regulation, and social motivation—domains that are often atypical in individuals with ASD [12,13,14]. As a result, adolescence may act as a tipping point where some individuals experience exacerbation of symptoms while others show compensatory adaptations or emerging strengths [15].

Given this background, the aim of the present paper is to provide a structured, integrative overview of the neurobiological and behavioral heterogeneity observed in adolescents with ASD, with particular emphasis on how developmental changes during adolescence intersect with the core and associated features of autism. Rather than offering an exhaustive review, we focus on a critical synthesis of current findings from neuroimaging, behavioral, and psychosocial domains to highlight key patterns, inconsistencies, and implications for intervention.

Our guiding question is as follows: How do neurodevelopmental changes during adolescence contribute to the variability in behavioral and cognitive outcomes observed in individuals with ASD? By addressing this question, we aim to clarify how adolescence both magnifies and reframes heterogeneity in ASD, and why a one-size-fits-all approach to diagnosis, support, or therapy is especially problematic during this life stage.

We further argue that understanding adolescent-specific trajectories is crucial not only for improving clinical care but also for informing models of neurodevelopmental plasticity and resilience. In the sections that follow, we examine evidence for structural and functional brain variability (Section 2), behavioral heterogeneity (Section 3), contextual influences including environmental and educational factors (Section 4), and implications for intervention and support (Section 5). We conclude by proposing future research directions that can better capture the dynamic and multifaceted nature of adolescence in ASD.

## 2. Neurobiological Heterogeneity in Adolescents with ASD

The neurobiological heterogeneity observed in adolescents with ASD reflects a complex interplay between genuine individual variability and methodological inconsistency across studies. Adolescence is a critical period of brain development marked by rapid changes in cortical thickness, white matter organization, and functional network architecture. Consequently, studies attempting to characterize neural differences in adolescents with ASD are especially vulnerable to confounding effects introduced by sample composition, imaging technique, preprocessing choices, and analytic design. The following section critically evaluates the main categories of neuroimaging evidence—structural, white matter, and functional—highlighting where findings converge, where they diverge, and how methodological heterogeneity contributes to inconsistent outcomes.

### 2.1. Structural Brain Findings

Neuroanatomical studies examining gray matter volume and cortical thickness in adolescents with ASD have yielded mixed and often contradictory findings. Some report increased cortical thickness in medial prefrontal regions and decreased volume in temporal poles compared to typically developing peers [12], while others observe no significant volumetric differences [13]. A major source of variability stems from the reliance of many early studies on region-of-interest (ROI) analyses using small, homogenous samples—typically high-functioning, male adolescents. These ROI-based studies are susceptible to confirmation bias, as they focus narrowly on regions hypothesized a priori to be implicated in ASD. In contrast, whole-brain voxel-based morphometry (VBM) approaches offer broader spatial coverage but introduce their own variability due to differences in segmentation algorithms, smoothing kernels, and thresholding strategies [14].

Moreover, the overwhelming majority of these studies are cross-sectional in design, limiting their ability to distinguish delayed maturation from deviant developmental trajectories. Longitudinal imaging, which would be more informative for tracking the evolving neuroanatomical phenotype of ASD, remains scarce in this age group. Compounding these limitations is the frequent omission or inconsistent control of key covariates such as IQ, co-occurring psychiatric conditions, and medication use. Together, these methodological and sample-related inconsistencies severely limit the comparability of structural findings across studies and may partially account for their divergent results.

### 2.2. White Matter Microstructure

Diffusion tensor imaging (DTI) studies investigating white matter integrity in adolescents with ASD have also reported highly variable findings, including both decreased and increased fractional anisotropy (FA) in major association tracts such as the corpus callosum, inferior longitudinal fasciculus, and uncinate fasciculus [15,16,17]. These discrepancies are not easily explained by underlying biological differences alone. Rather, they are likely influenced by differences in tractography methods—some studies employ tract-based spatial statistics (TBSS), while others use deterministic or probabilistic tractography. These techniques differ substantially in their sensitivity to crossing fibers, partial volume effects, and noise, affecting both the location and magnitude of reported group differences.

Another major confound in DTI studies is head motion, which is more prevalent in individuals with ASD and can systematically bias diffusion metrics if not rigorously controlled. While some studies report motion parameters and exclude participants with excessive movement, many do not, and fewer still apply advanced correction techniques such as outlier replacement or motion-scrubbing. This omission is particularly problematic given the known susceptibility of FA to motion-induced artifacts, especially in frontal and temporal tracts.

Sample size is also a critical concern. Many DTI studies in this population include fewer than 30 participants per group, resulting in low statistical power and inflated risk of false positives. Furthermore, the ASD population itself is extremely heterogeneous—encompassing a wide range of verbal ability, cognitive function, and adaptive skills—yet most studies do not stratify participants or account for this internal variability. As a result, the literature on white matter integrity in adolescent ASD remains fragmented and difficult to synthesize into a coherent narrative.

### 2.3. Functional Connectivity and Network Dynamics

Resting-state functional MRI (rs-fMRI) has been widely used to investigate large-scale brain network organization in ASD, yet findings remain inconsistent. Some studies report long-range hypoconnectivity, especially between nodes of the default mode network (DMN) such as the medial prefrontal cortex and posterior cingulate cortex [18], while others describe hyperconnectivity within salience or sensorimotor networks [19,20]. A key source of this inconsistency is the variability in preprocessing pipelines, particularly with regard to global signal regression (GSR). While GSR is intended to reduce physiological noise, its application can introduce spurious anti-correlations or invert the direction of observed group differences, leading to contradictory interpretations of the same underlying data [21].

Additional variability arises from differences in scan duration (ranging from 5 to 10 min), participant instructions (eyes open vs. closed), motion scrubbing thresholds, and spatial normalization templates. These choices, often left underreported, directly affect the reliability and replicability of functional connectivity measures. Moreover, many studies do not adequately account for individual differences in alertness, compliance, or cognitive engagement during scanning—factors that are especially variable in adolescents with ASD.

More recently, studies have employed dynamic functional connectivity (dFC) approaches to assess time-varying fluctuations in connectivity patterns. While this approach offers greater ecological validity, its implementation is highly inconsistent across studies. Sliding window lengths, clustering algorithms, and the number of identified connectivity states vary widely, making it difficult to determine whether reported abnormalities reflect true neural dynamics or methodological artifacts [22].

**Figure 1:**
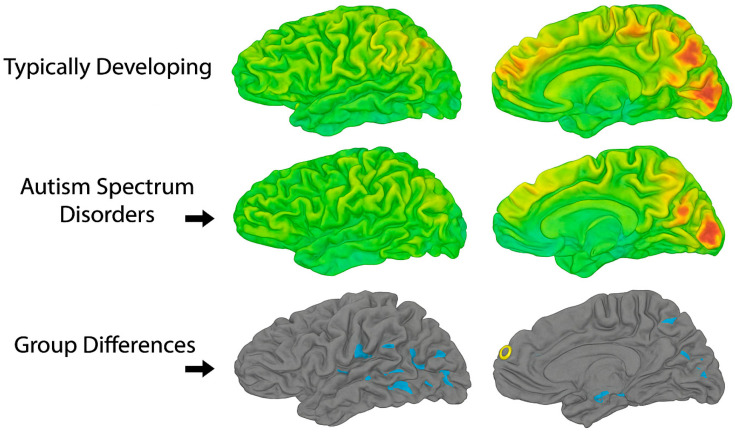
**Cortical surface maps comparing typically developing and autism spectrum disorder (ASD) groups**. Surface-based morphometry results are shown across lateral and medial cortical views. The top row (Typically Developing) and middle row (Autism Spectrum Disorders) display heat maps where color intensity represents the magnitude of the measured parameter (e.g., cortical thickness or functional activation), with green indicating lower values and yellow–red indicating progressively higher values. The bottom row (Group Differences) highlights regions showing statistically significant differences between groups, displayed on a gray cortical surface for contrast. Blue markings denote areas where ASD participants differ significantly from typically developing controls.

Furthermore, functional connectivity studies rarely stratify or statistically control for common ASD comorbidities such as ADHD or anxiety, despite clear evidence that these conditions independently alter functional network organization. Medication use—particularly of stimulants, SSRIs, or antipsychotics—is also frequently unreported or inconsistently excluded. These omissions further confound group comparisons and raise questions about the specificity of observed connectivity differences to ASD itself. “These connectivity patterns—characterized by relative underactivation in frontal control regions and overactivation in sensory-perceptual areas—are illustrated in Figure 1, which visually contrasts functional brain activity in adolescents with ASD versus neurotypical controls.”

The yellow areas demonstrate underactivity in prefrontal cortical regions, related to planning, complex cognition, decision-making, and adaptive behavior. The blue occipital and temporal regions are typically overactive in ASD, and at the top center, marked in blue, is a region that is likewise overactive and responsible for sensory processing.

### 2.4. Developmental Considerations and Methodological Standardization

A persistent limitation across neuroimaging studies in adolescent ASD is the tendency to pool participants into broad age categories, often spanning from late childhood (e.g., 10 years) through young adulthood (e.g., 19 years). This approach masks critical neurodevelopmental inflection points that occur during mid-adolescence, including synaptic pruning, myelination, and hormonal changes that influence social and cognitive processing. Given the neurobiological dynamism of this period, age-stratified analyses are essential to disentangle developmental effects from ASD-specific alterations.

To address the methodological challenges identified throughout this section, future research should prioritize the use of large, multisite datasets such as the Autism Brain Imaging Data Exchange (ABIDE) and ENIGMA-ASD, which facilitate replication and allow for the application of harmonized preprocessing pipelines. These collaborative efforts also support the use of machine learning and data-driven clustering techniques to identify neurobiological subtypes within ASD. However, such approaches will only be effective if grounded in robust data acquisition practices, transparent reporting standards, and careful phenotypic characterization. Standardization of scan protocols, motion correction strategies, and inclusion criteria—combined with stratification by age, IQ, comorbidities, and medication status—will be critical for advancing the field toward more interpretable and clinically relevant findings. These challenges are conceptually summarized in Figure 1, which illustrates how neuroimaging findings in adolescent ASD are shaped by both biological and methodological sources of heterogeneity.

## 3. Neurobiological Underpinnings of ASD in Adolescents

### 3.1. Brain Network Organization and Connectivity

#### 3.1.1. Functional Brain Abnormalities

**Figure 2:**
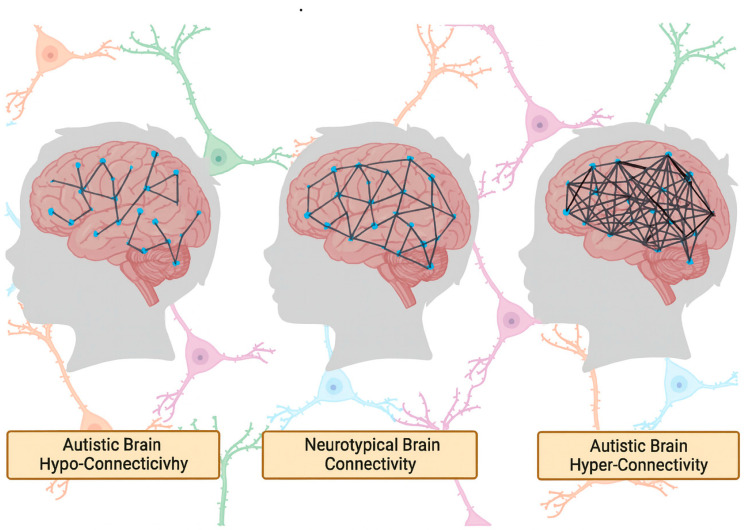
Functional connectivity deviations in ASD adolescents across core networks (DMN, SN, FPCN). Schematic representations of brain network connectivity are shown. Each brain image illustrates nodes (blue dots) representing cortical regions and edges (black lines) representing functional connections. The left panel depicts hypo-connectivity often reported in autism spectrum disorder (ASD), with fewer and weaker inter-regional con-nections. The middle panel shows neurotypical connectivity, characterized by a balanced pattern of network inte-gration. The right panel illustrates hyper-connectivity, also described in ASD, where excessive inter-regional con-nections may disrupt network efficiency. Colored neuronal images in the background are illustrative, symbolizing underlying neural substrates, and do not represent empirical data.

Neuroimaging studies have repeatedly demonstrated aberrant patterns of brain connectivity in individuals with ASD, particularly in adolescence, a time when the brain undergoes substantial reorganization. Functional magnetic resonance imaging (fMRI) and resting-state connectivity analyses have highlighted atypical inter-regional synchrony and developmental trajectory deviations in several networks, including the DMN, salience network, and frontoparietal control systems [23,24,25,26]. These findings have fueled the general hypothesis that ASD is associated with either underconnectivity or overconnectivity of brain networks [7]. These connectivity differences are exemplified in Figure 2.

However, the empirical foundation for these connectivity abnormalities is heterogeneous and sometimes contradictory. For example, Lawrence et al. [23] propose a developmental reconceptualization of functional connectivity in ASD, acknowledging that early overconnectivity may evolve into underconnectivity during adolescence, other studies, such as Haghighat et al. [26], point to mixed age-dependent hypo- and hyperconnectivity profiles across networks [25,26]. These divergent findings underscore the importance of methodological consistency, as variations in sample size, age grouping, imaging parameters, and analytical pipelines significantly shape outcomes.

For instance, some studies with smaller sample sizes (e.g., <30 ASD participants) lack statistical power to detect subtle network-level effects, leading to false positives or spurious group differences. Others rely on global signal regression or differ in motion correction thresholds—both of which are known to bias resting-state fMRI data, particularly in pediatric populations [27]. Such methodological discrepancies complicate cross-study comparisons and hinder efforts to delineate a reliable neurobiological signature of ASD.

Furthermore, task-based fMRI studies probing social cognition or executive function often use paradigms that vary in complexity and ecological validity. Some employ passive viewing tasks (e.g., facial affect recognition), while others use more demanding, multimodal social inference paradigms. The cognitive load and interpretive demands of these tasks differentially engage cortical and subcortical circuits, which may explain variability in reported activation patterns in regions such as the medial prefrontal cortex, posterior cingulate cortex, and temporoparietal junction [16,17,24].

A closer look at specific findings reveals additional methodological caveats. Lawrence et al. [23] reported that adolescents with ASD show atypical longitudinal development of functional connectivity, particularly in the DMN and sensorimotor networks. However, their longitudinal cohort was limited to high-functioning individuals, thus excluding a significant proportion of the ASD population with co-occurring intellectual disability or language impairments. This sampling bias reduces generalizability and may obscure distinct developmental trajectories among subgroups. Similarly, findings by Padmanabhan et al. [24] on DMN dysfunction rely heavily on spatial independent component analysis (ICA), a technique sensitive to the number of components extracted and potentially biased by group size disparities.

Adding further complexity, the distinction between intrinsic versus task-evoked connectivity patterns is rarely emphasized. Some studies conflate these by drawing overarching conclusions about network dysfunction from resting-state findings alone, despite the growing evidence that task-evoked modulations offer unique insights into cognitive inflexibility, social disengagement, or impaired self-referential processing in ASD [10,24,28,29].

Overall, the literature paints a compelling but methodologically fragmented picture of functional brain abnormalities in adolescents with ASD [30,31,32,33]. Future work must aim for greater harmonization of analytic pipelines, inclusion of diverse ASD subtypes, and standardized behavioral task batteries. Multi-site consortia using shared preprocessing pipelines (e.g., ABIDE, ENIGMA) [33] represent promising steps toward this goal, though even these initiatives face challenges related to sample representativeness, scanner variability, and phenotypic heterogeneity.

#### 3.1.2. Structural Brain Abnormalities

Structural neuroimaging studies in adolescents with ASD have revealed widespread alterations in brain morphology, yet these findings remain highly variable across individuals and studies. Commonly reported structural deviations include differences in cortical thickness, gyrification patterns, white matter integrity, subcortical volume, and minicolumnar organization [8,13,14,34,35,36,37]. However, the interpretation of such findings is often complicated by methodological inconsistencies and insufficient attention to sample heterogeneity.

**Figure 3:**
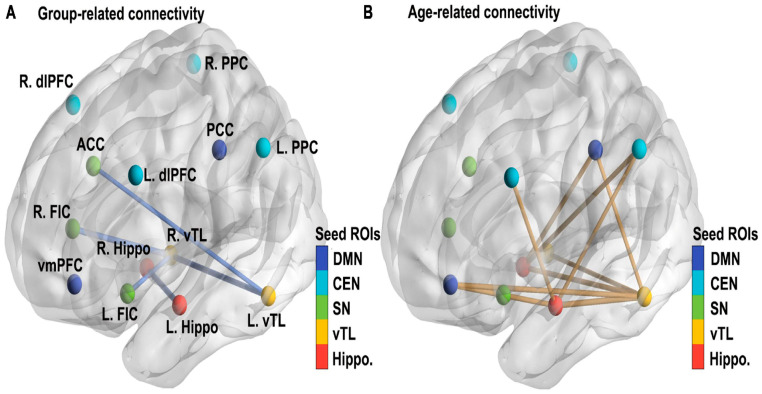
Group and Age Effects on Functional Connectivity Between Theory-Driven ROIs (from Chen L. et al [47]). (**A**) Significant group differences in functional connectivity among seed regions of interest (ROIs) derived from five literature-based networks: hippocampal memory, ventral temporal memory, salience, central executive, and default mode networks. All depicted connections show reduced connectivity in the ASD group compared to the non-ASD group. Line thickness corresponds to t-values from the regression model. (**B**) Age-related reductions in functional connectivity across the same theory-driven ROIs. (L/R dlPFC, left/right dorsolateral prefrontal cortex; PPC, posterior parietal cortex; ACC, anterior cingulate cortex; FIC, fronto-insular cortex; vmPFC, ventromedial prefrontal cortex; PCC, posterior cingulate cortex; hippo, hippocampus; vTL, ventral temporal lobe).

Several studies have documented region-specific cortical thinning in adolescents with ASD, particularly in temporal and parietal cortices, suggesting atypical synaptic pruning during adolescence [8,13]. Khundrakpam and colleagues [8] using a large-scale MRI dataset, reported that cortical thickness abnormalities in ASD persist across development and may shift in location and magnitude as individuals age. However, their cross-sectional design and use of non-uniform age bins limit their ability to capture true longitudinal trajectories. Furthermore, age-related changes in cortical metrics may reflect compensatory mechanisms or the influence of environmental and educational interventions, yet these factors are rarely controlled for. As illustrated in Figure 3, both diagnostic group and age significantly influenced functional connectivity among theory-driven ROIs spanning memory, salience, executive, and default mode networks, with adolescents with ASD exhibiting consistently reduced connectivity and age-related declines across these core circuits.

Similarly, volumetric studies have shown inconsistent findings. Schumann et al. [38] found enlarged amygdala volumes in children with ASD but not adolescents, while hippocampal enlargement persisted across age groups. Yet others, such as Zhang and associates [39], failed to replicate these volumetric patterns in the ABIDE dataset, potentially due to demographic or analytical differences. Moreover, voxel-based morphometry (VBM), a common approach in such studies, is highly sensitive to preprocessing choices, including tissue segmentation thresholds, spatial smoothing kernels, and registration templates [40]. These technical factors can significantly influence gray matter volume estimates and compromise reproducibility.

White matter abnormalities, particularly in association and commissural tracts, have also been frequently observed in adolescents with ASD. DTI studies report reduced FA in major tracts such as the corpus callosum, uncinate fasciculus, and superior longitudinal fasciculus, implicating disrupted connectivity between prefrontal and posterior cortices [41,42]. However, variations in DTI acquisition parameters (e.g., number of diffusion directions, b-values), tractography algorithms (deterministic vs. probabilistic), and motion artifacts, especially problematic in pediatric imaging, limit the comparability across studies [40,42]. Furthermore, findings from Andrews et al. [42] suggest that changes in white matter integrity may correlate with developmental changes in ASD severity, yet this relationship remains poorly understood and likely non-linear [42].

Other studies have proposed that neurodevelopmental deviations in ASD may stem from early overgrowth followed by arrested or regressive maturation. Courchesne et al. [43,44] presented compelling evidence of increased neuron number and cortical volume in young autistic children, particularly in the prefrontal cortex, followed by stagnation or reduction in adolescence. However, these findings are based largely on postmortem samples and structural MRI data from young children, with less clarity on how these early changes evolve during the adolescent period. Moreover, interindividual variability—driven by factors such as sex, IQ, comorbidities, and medication use—further complicates the interpretation of group-level averages.

Crucially, most studies of brain structure in ASD fail to stratify participants based on behavioral phenotype, symptom severity, or developmental trajectory. For instance, volumetric changes in the amygdala may differentially track with anxiety symptoms or social avoidance, yet such dimensions are often treated as nuisance covariates rather than key explanatory variables. The failure to account for such within-group heterogeneity may partially explain the contradictory findings in the literature and undermines efforts to draw reliable neuroanatomical conclusions about ASD as a whole [5,38,45,46].

To enhance interpretability and translational relevance, future studies must incorporate finer-grained phenotyping, employ harmonized structural imaging protocols, and prioritize longitudinal designs. Importantly, rather than searching for universal neuroanatomical biomarkers of ASD, researchers may be better served by identifying developmentally sensitive subtypes or trajectories of brain structure that map onto functional and behavioral profiles.

#### 3.1.3. Disruptions in the Default Mode Network and Social Brain Regions

Among the most consistently implicated systems in ASD are the DMN and the social brain, both of which undergo significant reorganization during adolescence. The DMN, comprising medial prefrontal cortex, posterior cingulate cortex, precuneus, and angular gyrus, is centrally involved in self-referential thought, mentalizing, and social cognition—domains frequently affected in ASD [24,25]. Numerous resting-state fMRI studies have reported reduced DMN coherence or hypoconnectivity in adolescents with ASD compared to neurotypical peers [17,23]. However, the magnitude and spatial distribution of these findings vary widely across investigations, raising important questions about methodological consistency and clinical interpretation.

Some of the heterogeneity in DMN findings may stem from task states (resting-state vs. task-based), age stratification, and participant characteristics such as verbal IQ, head motion, and medication status [17,40]. For instance, Padmanabhan et al. [24] demonstrated that disrupted DMN connectivity was more pronounced in adolescents with greater social impairment, suggesting a dimensional association rather than a categorical marker. However, many studies continue to treat ASD as a binary diagnostic entity, thereby neglecting clinically meaningful variation. Furthermore, while head motion is known to inflate long-range connectivity estimates and reduce short-range connectivity in fMRI analyses, only a minority of DMN studies in ASD rigorously control for motion artifacts, despite their heightened prevalence in this population.

The social brain, encompassing regions such as the superior temporal sulcus, amygdala, fusiform face area, and medial prefrontal cortex, also exhibits atypical development in ASD [16,48,49]. These areas are essential for processing biological motion, facial affect, and theory of mind—abilities that typically improve across adolescence but often remain impaired in ASD. Pelphrey et al. [16] have argued that variability in social brain network function may serve as a marker of heterogeneity in ASD, offering a path toward stratified models of the disorder. However, studies vary considerably in their operational definitions of “social brain” regions and their analytical strategies for connectivity mapping, rendering meta-analytic synthesis difficult.

Functional differences in the amygdala, a key hub of the social brain, have also been inconsistently reported. Some studies describe hyperactivation to social stimuli, while others observe hypoactivation, depending on the task context, participant anxiety levels, and developmental stage [50,51,52]. For example, Guo et al. [50] found decreased resting-state connectivity of the amygdala in adolescents with ASD, which was associated with greater social difficulties [50]. But their findings may be limited by relatively small sample size (N < 30 per group), lack of longitudinal data, and the absence of co-occurring psychiatric diagnoses assessment. Larger studies using multi-site data, such as those from the ABIDE dataset, have struggled to replicate localized findings, highlighting the need for harmonized imaging pipelines and cross-validated analytic models.

Despite these limitations, converging evidence suggests that DMN and social brain disruptions are not uniform across individuals with ASD but instead may reflect divergent neurodevelopmental trajectories. For instance, Wallace et al. [13] reported age-related cortical thinning in temporal and parietal DMN regions in adolescents with ASD, but the trajectory of thinning varied significantly depending on age bracket and functional outcomes. These developmental deviations may arise from altered synaptic pruning or myelination schedules, yet this remains speculative in the absence of multimodal longitudinal data. Furthermore, several studies ignore the potential impact of puberty—a phase of dramatic neurohormonal change—on brain connectivity, despite its likely relevance to social and emotional development in ASD.

Methodologically, the literature on the DMN and social brain in ASD is hampered by variability in region-of-interest definitions, signal denoising strategies, and statistical thresholds. These factors influence not only detection power but also the anatomical specificity of reported effects. A critical review by Nair et al. [17] underscored the importance of using developmentally appropriate templates and controlling for individual variability in brain maturation, especially when examining adolescent populations.

In future work, individualized connectome approaches and graph theoretical metrics may help capture the dynamic evolution of DMN and social brain organization in ASD. Furthermore, linking these neural profiles with behavioral subtypes, rather than global diagnostic categories, holds promise for elucidating the true sources of heterogeneity and identifying mechanistically meaningful subgroups.

### 3.2. Behavioral Heterogeneity in Adolescents with ASD

ASD is marked not only by neurobiological variability but also by profound behavioral heterogeneity, particularly during adolescence—a time of dynamic social, cognitive, and emotional change. While ASD is clinically defined by social communication deficits and restricted, repetitive behaviors (RRBs), the expression and severity of these domains vary significantly across individuals and over time [1,7,21,53]. This variability is further complicated by task design, assessment measures, and contextual factors, all of which must be critically considered when interpreting findings from behavioral studies.

#### 3.2.1. Social Communication Variability

Adolescents with ASD often demonstrate difficulties in pragmatic language, perspective-taking, and interpreting nonverbal cues. However, the degree and nature of these challenges are not homogeneous. Some individuals exhibit near-typical reciprocal conversation, while others remain largely nonverbal [1,54]. Furthermore, responses to social skills interventions vary considerably, with factors such as verbal ability, baseline motivation, and family support playing moderating roles. A recent meta-analysis by Cheng et al. [54] on UCLA PEERS^®^-based interventions reported significant heterogeneity in outcomes across trials, likely due to differences in inclusion criteria, session formats, and fidelity of delivery.

Task-related differences further cloud interpretation. Performance on standardized social cognition tasks (e.g., Theory of Mind, facial emotion recognition) often diverges from real-world social functioning. For example, adolescents may score within the normative range on lab-based tasks yet struggle with peer relationships in naturalistic contexts. These inconsistencies suggest a need for greater ecological validity in assessment design and interpretation.

#### 3.2.2. Repetitive Behaviors and Sensory Processing

RRBs and atypical sensory responses—ranging from hypersensitivity to hyposensitivity—are also highly variable across adolescents with ASD [48]. Some individuals exhibit intense preoccupations and insistence on sameness, while others display more subtle motor stereotypies. The manifestation of these behaviors often shifts with age, cognitive ability, and environmental stressors. For instance, stereotypies may decrease in frequency but increase in complexity during adolescence, while insistence on sameness may intensify during periods of high anxiety or transition.

Despite this, many studies categorize RRBs monolithically, overlooking nuanced distinctions between behavioral subtypes (e.g., circumscribed interests vs. motor mannerisms). Moreover, the prevalence and impact of sensory processing differences are often underreported or assessed using parent-report questionnaires with limited psychometric precision [48]. This methodological variability complicates efforts to link RRB phenotypes to underlying neural substrates, as different studies may operationalize the same construct in incompatible ways.

#### 3.2.3. Executive Function and Cognitive Flexibility

Executive function (EF) is another domain marked by considerable heterogeneity in ASD adolescents. Impairments in cognitive flexibility, working memory, and inhibitory control have been widely reported, yet these deficits are neither universal nor consistent across tasks [55,56]. Some individuals demonstrate profound difficulty with set-shifting (e.g., Wisconsin Card Sorting Task), while others perform comparably to typically developing peers. Task complexity, instructions, and prior exposure all influence performance, making inter-study comparison problematic.

Braden and associates [55] highlighted that EF impairments in middle-aged adults with ASD were significantly modulated by comorbid conditions and age-related decline, suggesting that developmental stage and broader mental health profiles must be considered when interpreting adolescent EF outcomes. Furthermore, Dajani and Uddin [56] argued that existing EF tasks often fail to capture the dynamic, context-dependent nature of cognitive flexibility in real-world settings [56].

Additionally, individual differences in motivation, stress response, and prior experience may shape EF performance as much as underlying neural circuitry. Thus, behavioral variability in EF should not be taken as a direct proxy for frontal lobe dysfunction without considering these moderating variables. Few studies stratify participants based on comorbidity (e.g., ADHD), even though such factors can strongly influence EF profiles.

#### 3.2.4. Emotional Regulation and Comorbid Psychopathology

Adolescence is a period of increased vulnerability to affective disturbances, and adolescents with ASD show elevated rates of anxiety, depression, and irritability. These emotional challenges contribute significantly to behavioral heterogeneity [7,57]. Importantly, the expression of emotion dysregulation can differ across sex, verbal ability, and environmental context. Some individuals exhibit outward expressions of anger and aggression, while others internalize symptoms, presenting with flat affect or withdrawal.

Goldman et al. [57] emphasized the role of sleep disturbance in modulating affective symptoms in adolescents with ASD, showing that disrupted sleep patterns correlate with increased irritability and emotional lability. However, many studies fail to control for sleep quality, medication use, or pubertal stage—factors that can meaningfully alter both emotional and behavioral expression.

Furthermore, the frequent co-occurrence of psychiatric diagnoses such as generalized anxiety disorder, OCD, or mood disorders introduces additional complexity. Some behavioral symptoms attributed to ASD may in fact reflect overlapping syndromes. For instance, rigidity and repetitive behaviors may stem from anxiety rather than core ASD traits, and affective flattening may reflect depressive symptoms rather than social disinterest. Without careful differential assessment, behavioral heterogeneity may be mischaracterized as intrinsic to ASD rather than due to comorbidity.

#### 3.2.5. Longitudinal and Contextual Considerations

Longitudinal studies reveal that behavioral profiles in adolescents with ASD are far from static. For example, Shattuck et al. [7] found that while core ASD symptoms showed modest decline from adolescence into adulthood, maladaptive behaviors such as irritability and hyperactivity often increased during the same period. These patterns suggest that developmental context—including school transitions, social demands, and hormonal changes—can shape the expression of ASD traits.

Nevertheless, many cross-sectional studies fail to account for these contextual variables, presenting a snapshot view that may mask intra-individual variability. Moreover, cultural and socioeconomic factors are rarely included in analyses, yet they likely modulate access to intervention, parental expectations, and behavioral expression.

In summary, behavioral heterogeneity in adolescents with ASD arises from a complex interplay of intrinsic, developmental, and environmental factors. Yet many studies inadequately address this complexity, relying on broad diagnostic categories and overly simplistic behavioral metrics. Future research should prioritize dimensional frameworks, multimodal assessment strategies, and longitudinal designs that can better capture the dynamic and individualized nature of adolescent ASD.

## 4. Default Mode Network and Cognitive Function in Adolescents with ASD

### 4.1. Atypical DMN Connectivity in ASD

The DMN, a prominent large-scale brain network encompassing the medial prefrontal cortex, posterior cingulate cortex/precuneus, and angular gyrus, is implicated in self-referential thought, social cognition, and internally directed processes. Aberrations in DMN connectivity have been repeatedly reported in ASD populations, with findings pointing to both hypo- and hyper-connectivity patterns across different developmental stages and clinical subgroups.

Padmanabhan et al. [24] employed resting-state fMRI to assess DMN function in children, adolescents, and adults with ASD. Their results indicated reduced long-range DMN connectivity in adolescents with ASD compared to age-matched controls. However, this study utilized a relatively small sample (N = 19 ASD adolescents), limiting generalizability. Additionally, the participants were not stratified by cognitive ability or symptom profile, obscuring whether the observed DMN disruptions were linked to specific subtypes or broader ASD characteristics. While their longitudinal design is commendable, more robust sample stratification and inclusion of behavioral correlates would improve interpretability. Furthermore, the use of seed-based correlation methods may have constrained the capacity to detect broader network-level disruptions or compensatory adaptations in adolescents with more intact cognitive functioning.

Lawrence et al. [23], using a larger and developmentally focused cohort, found that adolescents with ASD exhibit atypical developmental trajectories in DMN functional connectivity, particularly within the medial prefrontal and posterior cingulate cortices. The divergence from typical age-related connectivity maturation underscores the importance of studying ASD within a neurodevelopmental framework. Yet, this study’s reliance on cross-sectional rather than longitudinal data weakens the strength of inferences about within-individual change. Importantly, the authors did not control for the impact of psychiatric comorbidities (e.g., anxiety, ADHD), which are known to affect DMN function and are prevalent in adolescents with ASD, raising questions about the specificity of their findings to ASD per se.

In contrast, Nair et al. [17] provided a comparative review of DMN connectivity in adolescents with ASD and early-onset psychosis, revealing that both disorders exhibit disruptions in DMN integrity but with distinct topographical patterns. Their review critically synthesizes findings across multiple studies, though many of the included works employed varied preprocessing pipelines, ROI definitions, and head motion correction standards. These methodological inconsistencies make it difficult to determine whether reported DMN abnormalities reflect genuine disorder-specific alterations or analytic artifacts. Moreover, their review does not sufficiently address how developmental stage (early vs. mid-to-late adolescence) might moderate the nature or extent of DMN dysfunction.

These studies collectively highlight DMN abnormalities in adolescents with ASD, but methodological variability—including differences in preprocessing standards, parcellation schemes, and connectivity metrics—substantially limits cross-study comparability. Future investigations should adopt harmonized acquisition and analysis protocols, larger and phenotypically stratified samples, and developmental designs to discern which DMN alterations are truly diagnostic of ASD versus those reflecting developmental delay, comorbidity, or task-specific factors.

### 4.2. DMN and Social-Cognitive Impairments

Given the role of the DMN in social cognition, it is not surprising that aberrations in this network are frequently linked to social deficits in adolescents with ASD. Functional connectivity disruptions in the medial prefrontal cortex (mPFC) and posterior cingulate cortex (PCC) have been associated with reduced perspective-taking, impaired theory of mind, and diminished self-other differentiation.

Burrows et al. [29] reported that adolescents with ASD show decreased functional connectivity between the mPFC and temporoparietal junction (TPJ), two regions crucial for mentalizing. However, their findings were based on a limited cohort (N = 13 adolescents with ASD), and the study lacked direct behavioral correlations, rendering the clinical relevance of the connectivity alterations ambiguous. Additionally, the exclusive use of resting-state paradigms fails to capture how DMN nodes engage during active social tasks. A combined resting-state and task-based fMRI approach would provide a more nuanced understanding of how DMN dysfunction impacts social cognitive processes in this population.

Padmanabhan et al. [24], in the same cohort discussed earlier, also observed reduced coherence within the mPFC subnetwork during adolescence. Interestingly, in adults with ASD, some degree of normalization was seen, suggesting that DMN function may shift across developmental stages. However, their developmental interpretation is limited by the cross-sectional nature of the data, making it impossible to track within-subject neural trajectories. This introduces potential cohort effects and raises questions about whether observed “normalization” in adulthood reflects true recovery, compensatory reorganization, or sample bias (e.g., exclusion of more severely affected individuals due to compliance issues in scanning).

Moreover, studies like those by Dajani and Uddin [56] emphasize the need to conceptualize DMN function within a broader framework of network flexibility and integration. They argue that DMN abnormalities in ASD may not be isolated but part of a broader pattern of impaired switching between task-positive and task-negative networks, implicating systems such as the salience network and executive control network. Yet, the empirical grounding of this hypothesis remains limited, particularly in adolescents. Future work should integrate multi-network analyses and examine how atypical DMN dynamics relate to real-world social functioning over time.

### 4.3. Restricted and Repetitive Behaviors (RRBs)

RRBs are among the core diagnostic features of ASD and are highly heterogeneous in their manifestation during adolescence. These behaviors range from simple motor stereotypies to complex cognitive rituals and routines. While some individuals may exhibit persistent hand-flapping or rocking, others may demonstrate intense, narrow interests or rigid adherence to routines. The severity and type of RRBs can change over time, particularly during adolescence, influenced by both neurodevelopmental changes and social contexts [1,7,16].

Neuroimaging studies have suggested that RRBs may be linked to aberrant connectivity within cortico-striatal-thalamo-cortical circuits, including the basal ganglia and orbitofrontal cortex [4,9,10]. However, critical examination reveals methodological inconsistencies across studies. For example, differences in how RRBs are defined, measured, and categorized pose significant challenges. Some studies rely on parent-reported instruments (e.g., the Repetitive Behavior Scale-Revised), while others use clinician-administered tools or direct behavioral observations. These discrepancies contribute to variations in reported prevalence and neurobiological correlates of RRBs.

Age is another crucial moderating factor. The work of Uddin et al. [10] highlights that during adolescence, dynamic changes in brain plasticity may exacerbate or attenuate RRBs depending on contextual stimuli and environmental demands. However, findings remain mixed, with some longitudinal data indicating a reduction in lower-order repetitive behaviors (e.g., motor stereotypies) during adolescence, while higher-order behaviors (e.g., insistence on sameness, intense interests) may persist or even intensify [7,57].

Sample characteristics further complicate the landscape. Many studies examining RRBs in adolescents with ASD focus on high-functioning individuals, potentially neglecting more severe presentations. This bias may distort the generalizability of findings and reinforce stereotypes about RRBs being uniformly rigid or maladaptive. For example, some restricted interests may serve positive adaptive or coping functions in certain adolescents and thus warrant nuanced interpretation rather than pathologization.

In summary, while the neural correlates of RRBs are increasingly studied, the field suffers from inconsistent definitions, sampling bias, and lack of developmental specificity. A more rigorous stratification of subtypes, longitudinal designs, and harmonization of measurement tools are needed to disentangle the underlying neurobiology and developmental trajectory of RRBs in adolescence.

### 4.4. Sensory Processing Differences

Sensory processing atypicalities are frequently reported in adolescents with ASD and span hyperreactivity, hyporeactivity, and sensory-seeking behaviors across visual, auditory, tactile, olfactory, and proprioceptive modalities [48]. These differences are not merely peripheral phenomena but are central to the lived experience of many adolescents with ASD and often influence social, academic, and behavioral functioning [48,57].

Neuroimaging studies implicate atypical activity and connectivity in primary sensory cortices, the insula, and thalamic regions in mediating sensory processing differences [40,58]. For instance, thalamocortical dysconnectivity has been proposed as a neural signature underlying sensory filtering difficulties, with evidence of reduced integration between the thalamus and sensory association cortices [58]. Functional MRI studies also reveal abnormal activation in the insula and anterior cingulate cortex, suggesting disruptions in the salience network, which modulates attention to internal and external sensory stimuli [24].

However, a critical examination of the literature reveals notable methodological limitations. Many studies fail to differentiate between types of sensory symptoms (e.g., hyper- vs. hypo-responsiveness), instead aggregating them into composite scores that may obscure distinct neural correlates. Furthermore, there is wide variation in the instruments used to assess sensory reactivity, from parent- or self-report questionnaires like the Sensory Profile to clinician-based observational tools, leading to significant measurement heterogeneity. This inconsistency hampers cross-study comparisons and contributes to variable findings.

Another challenge relates to sample heterogeneity. Much of the literature focuses on either younger children or mixed-age samples, with fewer studies isolating adolescents as a distinct developmental group. Given that adolescence is marked by rapid neurobiological changes, including myelination and synaptic pruning in sensory and integrative brain areas, the developmental trajectory of sensory processing differences may be particularly volatile during this period [19,44]. Yet, only a handful of longitudinal studies track sensory symptoms from childhood through adolescence, leaving questions about their developmental stability unresolved.

Sensory sensitivities in adolescence may compound difficulties in social interaction, school participation, and emotion regulation. For example, auditory hypersensitivity may lead to social withdrawal or avoidance of crowded environments, thereby exacerbating social skill deficits [57]. However, relatively few studies explore these downstream functional consequences in depth. Moreover, the relationship between sensory symptoms and co-occurring anxiety or executive function difficulties remains underexplored, despite clinical evidence suggesting strong bidirectional influences.

To advance the field, future studies must adopt stratified methodologies that distinguish subtypes of sensory reactivity and link them to specific neural networks using well-powered, multimodal neuroimaging. In parallel, longitudinal designs are needed to assess the evolution of sensory symptoms and their predictive value for broader adaptive functioning.

### 4.5. Sleep Disturbances

Sleep disturbances are highly prevalent among adolescents with ASD, with estimates suggesting that between 40% and 80% of individuals experience some form of sleep dysregulation [57]. These disturbances include difficulty initiating and maintaining sleep, reduced total sleep time, night awakenings, and abnormal circadian rhythms. While sleep issues are often framed as a secondary concern, mounting evidence suggests they may exacerbate core ASD symptoms and significantly impact daytime functioning.

Functional and structural neuroimaging studies implicate several brain regions in sleep regulation abnormalities in ASD. These include the brainstem, hypothalamus, and pineal gland, with additional modulation by the thalamus and frontal cortex [40,57]. Melatonin dysregulation, often due to abnormalities in serotonin pathways, has been consistently observed, suggesting a neurochemical basis for delayed sleep onset and poor sleep maintenance.

Despite these findings, few studies have specifically focused on adolescents as a distinct developmental group. Most research either collapses across wide age ranges or includes only children, limiting the ability to disentangle age-specific neurobiological contributors. This omission is particularly problematic given that adolescence is a period of normative shifts in sleep architecture and circadian phase delay, which may interact with ASD-related neural atypicalities to compound difficulties [19].

Methodologically, studies investigating sleep in ASD vary considerably in terms of assessment tools. Objective measures like polysomnography and actigraphy are used inconsistently, and many studies rely solely on parent or self-report questionnaires, which are prone to bias and may not accurately capture sleep architecture. Sample sizes are often small and heterogeneous, and the inclusion of adolescents with comorbid psychiatric conditions (e.g., anxiety, ADHD) further muddies interpretation of findings. These factors together hinder the replicability and generalizability of results.

Furthermore, while several studies establish correlations between sleep quality and ASD symptom severity, few explore causality or directionality. For example, while disrupted sleep may exacerbate social-communication difficulties, it is also possible that anxiety related to social performance contributes to sleep onset insomnia—a bidirectional relationship that remains poorly understood.

Sleep disturbances in adolescents with ASD have been associated with impairments in attention, executive functioning, mood regulation, and adaptive behavior. Poor sleep may also amplify sensory sensitivities and contribute to irritability and aggression, underscoring its broad impact [57]. However, there is a dearth of intervention studies targeting sleep in adolescents specifically. Most behavioral sleep interventions have been developed for younger children, and pharmacological treatments such as melatonin are prescribed off-label with limited long-term efficacy data.

There is a pressing need for longitudinal and intervention-based studies that can clarify the mechanisms linking sleep and ASD features across adolescence. Given the heterogeneity of sleep profiles in ASD, stratified approaches—possibly guided by circadian biomarkers or neuroimaging—could help identify subgroups who may benefit from tailored interventions.

### 4.6. Emotion Regulation and Comorbidities

Emotion regulation (ER), the capacity to modulate emotional responses in accordance with contextual demands, is a key domain in which many adolescents with ASD experience profound challenges. Deficits in ER are not only intrinsic to ASD but also central to the development and maintenance of comorbid psychiatric conditions, most notably anxiety and depression. These challenges often manifest behaviorally as affective lability, heightened irritability, meltdowns, or social withdrawal, with significant implications for adaptive functioning and quality of life [55].

Neuroimaging studies have implicated altered connectivity within limbic circuits, particularly between the amygdala and prefrontal cortex, in emotion dysregulation among individuals with ASD [50,51,55]. The amygdala, responsible for emotion detection and salience processing, often shows hypo- or hyper-reactivity depending on the context, while the medial mPFC demonstrates inconsistent patterns of engagement across studies. This disrupted amygdala–PFC circuitry is thought to underlie deficits in emotional insight, empathy, and regulation.

However, studies vary considerably in sample size, age ranges, and methodology. For instance, Guo et al. [50] reported decreased amygdala functional connectivity in adolescents using resting-state fMRI, but their relatively small sample (n = 23 ASD; n = 21 controls) and the lack of task-based assessments limit the interpretability of findings. Other investigations, such as Braden et al. [55] employed both structural and functional imaging in middle-aged adults, raising concerns about age-related generalizability to adolescence. These discrepancies underscore the need for adolescent-specific, multimodal research that integrates structure, function, and behavior.

Anxiety disorders are among the most common comorbid conditions in adolescents with ASD, with prevalence estimates as high as 40% to 50% [54]. Anxiety often exacerbates social avoidance, leads to compulsive behaviors, and is strongly linked to intolerance of uncertainty—a trait observed across multiple ASD subgroups. Depression, although less frequently diagnosed in younger individuals with ASD, becomes increasingly prominent during adolescence and is associated with higher rates of suicidal ideation.

Studies examining these comorbidities often suffer from selection biases. Participants are typically high-functioning and verbal, potentially skewing estimates of prevalence and neural correlates. Additionally, many diagnostic instruments are not validated for ASD populations, especially when assessing internalizing symptoms, which may manifest atypically (e.g., as somatic complaints or increased repetitive behaviors).

Across studies on ER and comorbidity, heterogeneity in task paradigms and analysis pipelines complicates comparison. Emotion-processing tasks range from facial affect recognition to implicit regulation paradigms, with varying degrees of ecological validity. Furthermore, comorbid psychiatric conditions are often treated as exclusion criteria, eliminating individuals who may best represent the “real-world” complexity of ASD presentations. Few studies account for the potential confounding effects of medication use, pubertal status, or sleep disturbance, factors known to affect emotional processing. As such, claims regarding the universality or specificity of neural abnormalities in ER among adolescents with ASD must be viewed cautiously.

Future research should prioritize stratification based on ER profiles using dimensional approaches rather than categorical diagnoses. Neurobiologically informed subtyping, possibly through machine learning applied to neuroimaging data, could improve the predictive utility of findings and guide intervention. Furthermore, research should explicitly investigate how ER difficulties interact with other ASD features, such as sensory processing or executive dysfunction, to produce distinct behavioral phenotypes.

Interventions targeting ER, such as mindfulness-based programs, cognitive behavioral therapy, or biofeedback, have shown promise but remain understudied in adolescents with ASD. Incorporating insights from neurobiological studies into these interventions could enhance their specificity and efficacy. For example, neurofeedback protocols targeting amygdala–PFC connectivity patterns could provide novel avenues for remediation.

### 4.7. Social Cognition and Theory of Mind

Deficits in social cognition—including the ability to infer others’ beliefs, emotions, and intentions—are among the most salient and impairing features of ASD. These deficits, often conceptualized under the framework of Theory of Mind (ToM), become particularly pronounced during adolescence, when social demands intensify. While impairments in ToM are not universal in ASD, they are sufficiently common to warrant in-depth neurobiological and behavioral scrutiny [16,49,54].

Functional neuroimaging consistently implicates a network of brain regions associated with ToM, including the mPFC, TPJ, superior temporal sulcus (STS), and PCC [16]. In adolescents with ASD, hypoactivation in these regions during ToM tasks has been documented in several studies. For instance, Pelphrey et al. [16] found reduced activation in the STS and TPJ when individuals with ASD attempted to infer the intentions of others. Sato et al. [49] similarly reported diminished gray matter volume in the so-called “social brain” network, suggesting potential structural underpinnings of social cognition deficits.

While such findings are compelling, several methodological caveats warrant attention. Sample sizes in many studies are modest (often <30 per group), limiting statistical power and inflating the risk of both false positives and false negatives. Furthermore, most tasks used to assess ToM rely on artificial or static stimuli, such as cartoon stories or still images, which may fail to capture the dynamic and context-dependent nature of real-world social interactions.

Age-related variability is another critical concern. Some studies pool data from children, adolescents, and adults, obscuring developmental nuances. For example, findings in younger children may reflect delayed maturation of social brain networks, whereas findings in adolescents may represent developmental divergence. Additionally, sex differences in ToM processing—which may be particularly relevant in light of the proposed “female autism phenotype”—are rarely considered in analyses, even though females with ASD may show more subtle ToM deficits or use compensatory strategies.

ToM tasks are often cognitively demanding, requiring not only social inference but also executive functioning, working memory, and language comprehension. Consequently, it can be difficult to disentangle core ToM deficits from more general cognitive limitations. This issue is especially relevant in samples that include individuals with intellectual disability or language impairments. Failure to control for these variables leads to heterogeneity in findings and reduces the specificity of conclusions.

Additionally, fMRI studies assessing ToM are often task-based and cross-sectional, which limits inferences about causality or developmental trajectories. Few studies examine how changes in social cognition relate to evolving brain connectivity or structural development over time.

It is also important to note that some adolescents with ASD exhibit near-typical performance on ToM tasks despite atypical neural activation patterns. This has led to the hypothesis that compensatory mechanisms—such as increased reliance on executive control or rote memorization of social rules—may support behaviorally normative performance. However, these strategies may be brittle and less effective in real-life, unstructured contexts.

Studies like that of Braden et al. [55] support this notion by showing preserved performance despite atypical activation in medial prefrontal and temporal regions during social-cognitive tasks. However, these compensatory routes have not been systematically mapped, and their neural basis remains poorly understood.

To address these limitations, future research should include larger and more diverse samples, use ecologically valid paradigms (e.g., video-based or interactive tasks), and apply longitudinal designs to track developmental trajectories. Stratification by sex, IQ, and co-occurring conditions will be essential to tease apart subgroup-specific patterns. Moreover, integrating neuroimaging with behavioral interventions may allow identification of biomarkers predictive of treatment response.

Understanding the neural and cognitive underpinnings of ToM deficits is critical for developing targeted interventions. Social cognition training programs, such as those based on the UCLA PEERS^®^ model, have demonstrated modest improvements in social functioning [54]. However, individual differences in neurobiological substrates may moderate treatment outcomes—a hypothesis that remains to be systematically tested.

### 4.8. Executive Function and Adaptive Behavior

Executive function (EF) encompasses a set of higher-order cognitive processes that support goal-directed behavior, including planning, working memory, cognitive flexibility, inhibition, and attentional control. These abilities are crucial for everyday adaptive functioning, especially during adolescence—a developmental window marked by growing demands for autonomy, organization, and emotional regulation. In adolescents with ASD, deficits in EF are among the most consistently observed cognitive impairments [55], and they strongly predict challenges in academic achievement, social competence, and independent living skills [59].

EF is supported by a distributed network of brain regions centered on the PFC, including dorsolateral and ventromedial regions, as well as the anterior cingulate cortex (ACC) and parietal lobes. In individuals with ASD, neuroimaging studies have reported hypoactivation in these regions during EF tasks, along with atypical structural connectivity between the PFC and other cortical and subcortical regions [55]. For instance, Braden and colleagues [55] documented both structural and functional abnormalities in frontal systems in middle-aged adults with ASD, which may reflect persistent developmental divergence originating in adolescence [55].

However, many studies of EF in ASD do not isolate adolescents as a discrete developmental group. This is a significant limitation, as adolescence is associated with a normative increase in PFC myelination, synaptic pruning, and connectivity reorganization [19,60]—processes that may be altered in ASD. Thus, findings from adult or pediatric populations may not generalize to adolescents, and studies that conflate these age groups risk obscuring critical developmental trajectories. The methodological heterogeneity in EF research is substantial. Studies use a wide range of cognitive tasks (e.g., Wisconsin Card Sorting Test, Tower of London, Stroop tasks), each tapping different EF subdomains. Performance on these tasks is also influenced by motivational factors, anxiety, and familiarity with test formats. Additionally, test–retest reliability for many EF tasks is modest, and ecological validity is often low, raising questions about the generalizability of lab-based findings to real-world behaviors.

Sample sizes in neuroimaging studies of EF in ASD are frequently small, and the inclusion criteria vary widely. Some studies include only high-functioning individuals, while others fail to control for comorbid ADHD, which itself is associated with EF deficits. As a result, effect sizes are inconsistent, and replication is limited.

While cognitive EF tasks assess abstract problem-solving abilities, adaptive behavior refers to the real-world execution of everyday tasks such as managing time, hygiene, communication, and social interaction. Adolescents with ASD often exhibit a striking dissociation between intellectual ability and adaptive behavior, a phenomenon known as the “IQ–adaptive behavior gap” [59]. This gap may reflect impairments in EF, as well as difficulties in generalizing learned skills to novel or unstructured environments.

Yet, few studies integrate direct EF assessments with standardized measures of adaptive behavior (e.g., Vineland Adaptive Behavior Scales or the ABAS-II). Moreover, the neurobiological mechanisms linking EF deficits to adaptive outcomes remain underexplored. While Braden et al. [55] and others have noted that EF deficits may be associated with reduced integrity of frontoparietal networks [55], these associations have rarely been tested longitudinally.

The trajectory of EF development in ASD is heterogeneous. Some adolescents show gradual improvement in EF skills, while others exhibit persistent or worsening difficulties. Longitudinal studies such as Andrews et al. [42] have begun to chart white matter development in relation to ASD symptomatology, but few have specifically linked these findings to EF or adaptive skill growth.

Factors contributing to this variability include sex, pubertal timing, environmental support, and intervention history. Additionally, cultural and socioeconomic factors may shape how EF and adaptive behavior are expressed and evaluated, yet these are often ignored in study designs.

Future studies should prioritize multi-method, longitudinal designs that integrate neuroimaging, cognitive testing, and adaptive behavior ratings. Stratified sampling based on EF profiles or trajectories could help identify subgroups with distinct support needs. Moreover, task-based fMRI studies should incorporate ecologically valid paradigms that simulate real-life executive demands.

Targeted EF interventions, such as those focusing on cognitive flexibility or time management, may yield substantial improvements in adolescents’ ability to function independently. However, few intervention studies to date have included neurobiological outcome measures, making it difficult to assess mechanistic change. Bridging this gap between brain and behavior will be critical for developing personalized, neuroscience-informed approaches to intervention.

### 4.9. Implications for Education and Social Integration

Adolescents with ASD face considerable challenges in educational and social contexts, particularly during the transition from structured primary school environments to the more demanding and less scaffolded settings of secondary education and beyond [1,21,48]. These challenges are deeply intertwined with the neurobiological and cognitive heterogeneity documented in previous sections and are further modulated by contextual factors such as classroom environment, educator training, and the availability of peer supports.

Academic achievement in adolescents with ASD is highly variable and not reliably predicted by IQ alone [21]. Some individuals perform at or above grade level, while others struggle with even basic literacy or numeracy. Cognitive heterogeneity, particularly in executive function, working memory, and processing speed, likely contributes to these disparate outcomes [55,56]. For instance, adolescents with intact language skills but poor cognitive flexibility may succeed in structured academic tasks yet falter in open-ended problem-solving or collaborative learning environments.

Unfortunately, educational assessments often fail to differentiate between core academic ability and the executive or attentional factors that may impede performance. Moreover, many studies that report on educational outcomes in ASD rely on caregiver or teacher reports rather than objective academic measures. This methodological limitation complicates efforts to identify specific neurocognitive predictors of academic success.

The adolescent period brings increasing emphasis on peer relationships, group belonging, and social identity formation. Adolescents with ASD are at elevated risk of social isolation, bullying, and mental health difficulties arising from poor peer integration [1,21,54]. These difficulties are not solely a function of social cognition deficits (as discussed in Section 4.6), but are also shaped by environmental factors such as classroom inclusion policies, peer attitudes, and educator expectations.

The literature evaluating social outcomes in adolescents with ASD is heterogeneous. Many studies use broad parent-report scales that conflate quantity of social interaction with quality or satisfaction. Others focus on observable behaviors without accounting for internal social motivation, which may be intact even in adolescents with marked communication difficulties. Consequently, the extent to which neurobiological differences translate into social exclusion remains difficult to quantify.

Intervention programs aimed at improving social functioning, such as the UCLA PEERS^®^ curriculum, have shown promise in enhancing social communication and peer interaction skills in adolescents and young adults with ASD [54]. However, findings are inconsistent, and effect sizes vary widely across studies. One limitation is the short duration and limited generalizability of these interventions. Most rely on structured, clinician-led sessions and do not sufficiently address the transition of skills into unstructured, peer-driven environments such as lunchrooms, sports teams, or online platforms.

Furthermore, few intervention studies stratify participants based on neurobiological profiles, such as functional connectivity patterns or EF impairments. This limits the ability to determine which adolescents are most likely to benefit from specific programs. For instance, those with prominent amygdala-prefrontal dysconnectivity may struggle with emotion regulation in social situations, requiring different support than individuals with intact affective responses but poor planning or inhibition [24,50].

From a policy perspective, inclusion models that emphasize neurodiversity and individualized supports have gained traction, but implementation remains inconsistent. Educators often lack training in ASD-specific learning profiles, and school systems may not provide sufficient resources for one-on-one support, social skills coaching, or executive function accommodations.

A further complication is the relative scarcity of longitudinal studies assessing educational and social integration outcomes in adolescents with ASD. Without such data, it is difficult to determine the long-term impact of specific supports or identify critical transition points that require targeted intervention.

Research must move toward identifying neurocognitive and behavioral subtypes that predict differential responses to educational and social interventions. Multi-site, longitudinal studies with harmonized measures of academic performance, social functioning, and neurobiological change are urgently needed. Moreover, the inclusion of autistic voices in shaping research questions and educational practices will be essential to ensure ecological validity and ethical alignment.

Interdisciplinary collaboration between neuroscientists, educators, psychologists, and policy-makers is critical to translating complex findings into practical classroom strategies. This includes creating individualized education plans (IEPs) that are informed not only by test scores, but by profiles of cognitive flexibility, sensory sensitivity, and social motivation.

## 5. Behavioral Phenotypes and Clinical Heterogeneity

Behavioral heterogeneity in adolescents with ASD manifests across multiple domains including communication, social interaction, emotional regulation, sensory responsiveness, and executive functioning. This section explores the complexity of these behavioral expressions and their neural underpinnings, emphasizing how methodological variability across studies may influence observed patterns and interpretations.

### 5.1. Communication and Social Interaction

Communication and social deficits are core diagnostic features of ASD, yet their expression varies widely among adolescents. While some individuals demonstrate profound impairments in verbal and nonverbal language, others may present with fluent but pragmatically atypical speech. These variations are influenced by factors such as intellectual functioning, comorbidities, and environmental exposure to social learning opportunities.

**Figure 4:**
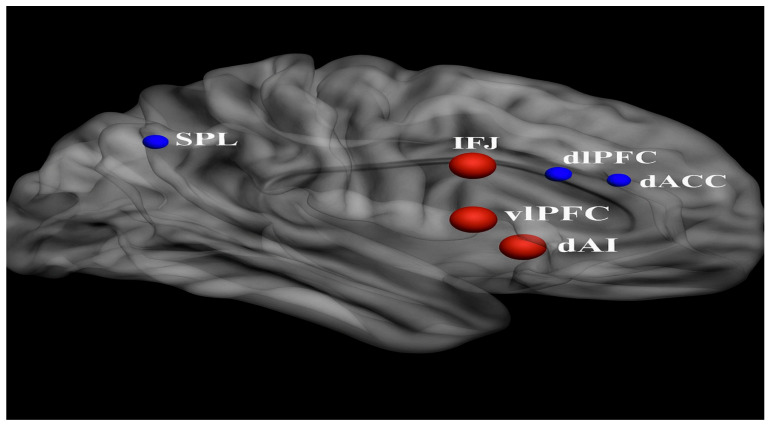
Regions implicated in social cognition dysfunction in adolescents with ASD. Areas in bule and red denote reduced gray matter volume and functional hypoactivation in regions such as the medial prefrontal cortex (mPFC), posterior superior temporal sulcus (pSTS), and temporoparietal junction (TPJ). These findings are representative of aggregated results from voxel-based morphometry and functional MRI studies [16,49].

For example, studies reporting on social brain networks in ASD have highlighted reduced activation and connectivity in key regions such as the superior temporal sulcus and medial prefrontal cortex during tasks requiring social cognition [16,49]. However, the extent of hypoactivation and its behavioral correlates vary significantly across studies. This discrepancy may stem from methodological differences, including the use of passive viewing versus interactive tasks, small sample sizes, and differing age ranges or IQ matching protocols. Neuroimaging findings have repeatedly implicated key regions within the social brain, including the mPFC, posterior superior temporal sulcus (pSTS), TP), in adolescents with ASD, with consistent evidence of reduced gray matter volume and functional hypoactivation in these areas represented in Figure 4.

Pelphrey et al. [16] attempted to constrain heterogeneity by focusing on developmentally sensitive measures of social brain function, but the sample was limited in size and lacked adequate representation of adolescents across the full spectrum of verbal abilities. Similarly, Sato et al. [49] reported reduced gray matter volume in social cognition-related regions, yet their cross-sectional design limits conclusions about developmental trajectories. Future work should prioritize longitudinal designs with larger and demographically diverse samples to elucidate how social communicative behaviors evolve in relation to brain maturation.

### 5.2. Emotional and Sensory Regulation

Emotional dysregulation and atypical sensory processing are frequently co-occurring challenges in adolescents with ASD. Clinical reports often describe heightened reactivity to sensory input, difficulties in modulating affect, and elevated rates of anxiety. These phenomena, however, are not uniformly expressed and appear to have neurobiological correlates.

DuBois et al. [48] reviewed various tools used to assess sensory dysfunction in adolescents and adults with ASD, but the heterogeneity in measurement approaches limited the comparability of results. Differences in parent-report versus self-report instruments, as well as inconsistency in operationalizing “sensory reactivity,” raise questions about the generalizability of findings. Furthermore, the study samples often included both adolescents and adults, with limited stratification by age or symptom severity.

On the neurobiological front, studies have linked hyperactivity in limbic circuits, including the amygdala, with both emotional reactivity and sensory sensitivity [5,50,51]. Yet these associations are often correlational and fail to account for important confounders such as medication status or comorbid psychiatric diagnoses. Guo et al. [50,51], for instance, found reduced amygdala connectivity in adolescents with ASD, but their analyses did not distinguish between participants with sensory sensitivity versus those without. This raises the possibility that the reported neurobiological signatures may reflect subgroup-specific effects that are not captured in group-level comparisons.

A more nuanced understanding of how emotional and sensory dysregulation intersect, and how they are mediated by underlying brain circuits, requires studies that incorporate deep phenotyping alongside neuroimaging, ideally in well-characterized subgroups stratified by sensory profiles or anxiety levels.

### 5.3. Executive Functioning and Adaptive Behavior

Executive dysfunction is another domain in which heterogeneity is pronounced. Commonly reported issues include deficits in cognitive flexibility, working memory, and goal-directed planning. However, performance on executive function tasks varies widely across individuals with ASD, even within age- and IQ-matched groups.

Braden et al. [55] used both structural and functional neuroimaging to investigate executive function in middle-aged adults with ASD, finding altered connectivity in frontoparietal and cerebellar networks. While these findings are instructive, their applicability to adolescents is limited due to developmental differences in executive circuitry maturation. Moreover, the sample size was small and included only high-functioning individuals, introducing selection bias.

Uddin [10] proposed that flexible cognition in ASD may be constrained by atypical developmental trajectories of control networks. However, this conceptualization has yet to be rigorously tested in adolescent samples. Cross-sectional studies with heterogeneous task demands make it difficult to parse whether observed deficits are due to core executive dysfunction, task-related confounds, or broader developmental delays.

Additionally, adaptive behavior, a real-world expression of executive capacity, often lags behind cognitive ability in ASD. Smith et al. [59] showed that daily living skills plateau or decline in adolescence, even among those with average IQ. The lack of standardization in how adaptive behavior is assessed across studies (e.g., Vineland vs. other instruments) further complicates synthesis of findings and identification of reliable brain-behavior correlates. 

## 6. Behavioral Correlates of Neurobiological Heterogeneity

### 6.1. Executive Functioning in Adolescents with ASD

Executive dysfunction is one of the most consistently reported behavioral features in ASD, encompassing deficits in planning, impulse control, attentional shifting, and working memory. Adolescents with ASD often demonstrate reduced cognitive flexibility, contributing to the rigidity and stereotyped behavior patterns typical of the condition [10,56]. Neurobiologically, these behaviors have been associated with abnormalities in the prefrontal cortex and its interconnections with the basal ganglia and cerebellum. Uddin et al. [10] argue that aberrant brain network dynamics underlie these behavioral impairments, with failure to transition between brain states as a marker of cognitive inflexibility.

However, the operationalization of executive function varies substantially across studies, and so does the selection of tasks used to measure it. For example, Stroop-like tasks versus spatial working memory paradigms may engage partially overlapping yet distinct neural circuitry. Thus, reported correlations between network connectivity and executive behavior should be interpreted with caution, as methodological differences may account for variability in findings across samples [56].

Moreover, most studies investigating executive functioning in ASD rely on cross-sectional data, limiting insights into developmental trajectories. Age-related maturation of prefrontal and frontoparietal systems in adolescents with ASD is still not well understood, and longitudinal data remain sparse. These gaps complicate efforts to disentangle true neurodevelopmental divergence from delays in maturation [20,42].

### 6.2. Social Cognition and Theory of Mind

Social deficits remain core diagnostic features of ASD. Impairments in ToM, the ability to attribute mental states to others, are particularly salient in adolescents as peer relationships become more complex. Neuroimaging studies have identified hypoactivation in the mPFC, posterior superior temporal sulcus pSTS, and TPJ in adolescents with ASD during social reasoning tasks [16,24,49]. These findings align with the concept of a “social brain” network that develops atypically in ASD.

While the presence of hypoactivation in these regions is widely reported, the magnitude and localization of effects vary significantly across studies. Inconsistencies may stem from variation in task design (e.g., false-belief tasks vs. emotional attribution), participant age, and IQ. For instance, high-functioning individuals with ASD may engage compensatory mechanisms, masking expected deficits in fMRI studies [16,24].

Some researchers have also questioned whether the observed neural signatures represent deficits in social cognition per se, or more general difficulties with complex inferential processing. The lack of specificity in the tasks used makes it difficult to draw direct causal links between neural findings and behavioral ToM profiles. Additionally, few studies adequately control for co-occurring conditions such as anxiety or ADHD, which may further impact social cognitive performance [7,21,57].

### 6.3. Neural Correlates of Restricted and Repetitive Behaviors in ASD"

RRBs in adolescents with ASD include insistence on sameness, restricted interests, and sensory hypersensitivities. Neurobiologically, these behaviors have been linked to dysregulation in corticostriatal circuits, particularly involving the orbitofrontal cortex, striatum, and thalamus [4,26,58]. Disrupted connectivity between these regions has been proposed as a mechanism for the generation and maintenance of repetitive behaviors.

The strength of these conclusions is tempered by several methodological limitations. Task-free (resting-state) studies, while valuable, may not capture the functional dynamics associated with actual RRB performance. Moreover, behavioral phenotyping of RRBs is often coarse, relying on caregiver report or broad clinical scales that do not differentiate between types of RRBs (e.g., motor stereotypies vs. cognitive rigidity). This lack of behavioral precision limits the interpretability of imaging findings [4,48].

Furthermore, sample sizes in imaging studies of RRBs are typically small, and the effect sizes reported vary widely. Some studies suggest hyperconnectivity in motor and sensory areas, while others report hypoconnectivity, likely reflecting developmental stage, analysis pipeline, or even scanner-specific artifacts [48,58]. Given these inconsistencies, more standardized task protocols and larger sample cohorts are necessary for replicable conclusions.

## 7. Transition to Adulthood

### Developmental Changes from Adolescence to Adulthood

Development from adolescence to adulthood in individuals with ASD is characterized by both neural and behavioral trajectories that diverge from those observed in NT peers. During this period, the maturation of brain function and structure, as well as the acquisition of adaptive skills, often follows an atypical course in ASD, with significant implications for adult outcomes. Neuroimaging studies reveal that the patterns of neural activation associated with cognitive tasks, such as face and object recognition, evolve differently in ASD compared to NT individuals. In NT adolescents, the similarity of activation patterns for within-category exemplars, such as faces, increases with age, reflecting a refinement of neural representations. This is evidenced by rising representational similarity analysis scores from adolescence to adulthood, indicating that neural coding becomes more specialized and efficient over time. However, this developmental increase in neural similarity is not observed in individuals with ASD, suggesting a disruption in the typical maturation of category-specific brain networks.

O’Hearn and Lynn [19] indicate that this divergence becomes particularly pronounced in adulthood, where group differences in neural activation patterns are evident. However, their study design was cross-sectional rather than longitudinal, which limits the ability to draw firm conclusions about developmental trajectories. Additionally, their participant pool, while informative, was relatively small and focused on high-functioning individuals, raising concerns about generalizability across the broader ASD spectrum. These limitations underscore the need for more longitudinal studies with larger and more diverse samples to validate the findings.

Behaviorally, these neural differences are mirrored in the progression of cognitive and adaptive skills. For instance, while both NT and ASD groups show improvement in face recognition from childhood to adolescence, only NT individuals continue to improve from adolescence into adulthood. In contrast, individuals with ASD exhibit a plateau in both face and car recognition abilities during this transition, highlighting a stagnation in the development of certain cognitive functions. However, most of these results stem from standardized behavioral tasks that may not fully capture functional performance in real-world settings. More ecologically valid assessments are required to determine whether these plateaus persist in naturalistic contexts. This plateau is not limited to recognition tasks; it extends to broader domains such as emotional self-awareness, daily living skills, executive function, and social cognition.

The lack of continued improvement in these areas during adolescence and early adulthood in ASD stands in contrast to the ongoing developmental gains seen in NT peers [19]. The neural underpinnings of these behavioral patterns are further elucidated by studies examining white matter development. A substantive and pervasive decline in cortical white matter development occurs in ASD during childhood and adolescence, potentially offsetting initial brain enlargement and contributing to the normalization of brain volume disparities relative to NT controls. However, this reduction in white matter growth could also underlie the arrested or atypical development of cognitive and adaptive skills observed in ASD during the transition to adulthood [61]. Notably, many of these studies use different tract-based or voxel-based approaches with variable thresholds for defining white matter integrity, introducing potential inconsistencies in reported findings. Additionally, motion artifacts and differences in acquisition protocols may confound group comparisons in younger and older cohorts.

Increases in fractional anisotropy within the cingulum, a marker of white matter maturation, are typically seen from childhood to adulthood, but such changes may be attenuated or altered in ASD, particularly in tracts adjacent to regions implicated in social cognition, such as the temporoparietal junction [24]. Yet, many studies examining this tract focus on adolescents and young adults with average or above-average IQ, limiting insight into developmental variability across cognitive subgroups. The trajectory of daily living skills provides a clear example of these developmental differences.

While some improvement in adaptive behaviors is observed from childhood to adolescence in ASD, these gains often plateau during adolescence and early adulthood, resulting in persistent challenges with independence and self-care [19]. Smith et al. [59] outline that the presence of intellectual disability is associated with both lower initial levels and slower growth in daily living skills, further compounding difficulties during the transition to adulthood. Their longitudinal design strengthens the findings, but the use of parent-report measures may introduce biases related to caregiver expectations or interpretation of abilities. The rate of change in these skills tends to decrease over time, and although age is associated with initial skill levels, the expected curvilinear improvement seen in NT individuals is often absent or diminished in ASD. This highlights the importance of stratifying analyses by cognitive level and baseline functioning in future longitudinal research.

Levy and Perry [21] note that raw scores on cognitive and adaptive measures may increase more slowly than chronological age, reflecting a plateau rather than a decline in abilities. However, their synthesis draws heavily from studies conducted in Western, clinical settings, raising questions about cultural variability in developmental expectations and support systems. The period of adolescence itself is marked by increased social demands, the onset of pubertal maturation, and a shift in behavioral and emotional regulation. These changes are particularly challenging for individuals with ASD, who may experience heightened difficulties in adapting to new expectations and environments [50]. The interplay between neural maturation, cognitive development, and environmental demands underscores the complexity of the transition from adolescence to adulthood in ASD. Collectively, these findings emphasize that the developmental trajectory from adolescence to adulthood in ASD is characterized by a combination of arrested or atypical neural maturation, stagnation in cognitive and adaptive skill acquisition, and persistent challenges in social and behavioral functioning. Understanding these patterns is essential for designing interventions that address the unique needs of adolescents and young adults with ASD as they navigate the transition to greater independence [19,50,59,61].

## 8. Discussion

Adolescents with ASD face multifaceted challenges during their transition to adulthood, encompassing neural, behavioral, social, and adaptive domains. These difficulties are closely linked to core ASD characteristics, including social communication deficits, restricted and repetitive behaviors, and impaired adaptive functioning [1,19]. While current research has made substantial progress in delineating neurobiological and behavioral heterogeneity, the developmental implications of these findings for adolescents remain inadequately addressed in the literature. This section synthesizes current evidence while critically evaluating methodological limitations, conflicting data, and unanswered questions specific to adolescents with ASD.

A central issue is the generalization of learned skills to novel environments such as postsecondary education and employment. Although this transition is expected in both ASD and NT adolescents, individuals with ASD often struggle with flexibility, social inference, and executive functioning, all of which impair skill transfer. However, much of the literature lacks clarity on whether these difficulties stem from persistent neurocognitive deficits, insufficient instructional support, or systemic educational shortcomings. Tailored transition planning is essential, but research has yet to determine which components of such programs are most effective for diverse ASD profiles.

Volkmar et al. [1] emphasize the importance of coordinated efforts between families, educational institutions, and adult service providers. However, studies often neglect to evaluate the fidelity of such collaborative models or control for variability in socioeconomic status and access to services. Additionally, most outcome studies rely heavily on self-report or caregiver report, raising concerns about subjective bias and the lack of objective functional outcome measures.

Family support remains indispensable during this period, especially in contexts lacking public resources. Yet, the literature seldom accounts for cultural variations in family involvement or differences in caregiver burden across the ASD spectrum. There is a clear need for studies assessing how different family structures, caregiving styles, and support systems influence transition outcomes.

The plateau in daily living skills during adolescence and early adulthood, as described by O’Hearn et al. [19], is well documented, yet the mechanisms remain equivocal. Is this stagnation primarily neurobiologically driven—due to halted white matter development—or is it a consequence of inadequate skills training or low expectations from caregivers and educators? Longitudinal studies with multimodal assessment (e.g., combining neuroimaging with ecological momentary assessment) could clarify this.

Social outcomes also show marked variability. While some individuals form romantic relationships and achieve independence, others remain socially isolated. Unfortunately, methodological heterogeneity—ranging from differing diagnostic criteria to nonstandardized outcome measures—hampers cross-study comparisons. Further complicating interpretation is the frequent underrepresentation of females and individuals from racially and ethnically diverse populations in these studies, limiting generalizability.

On the neural level, altered connectivity patterns, particularly involving the amygdala, are repeatedly implicated in ASD-related social and emotional regulation deficits [23,50]. However, developmental trajectories remain unclear due to the predominance of cross-sectional rather than longitudinal designs. Moreover, the relationship between neural patterns and functional behavior is often assumed rather than directly tested, leaving gaps in causal inference.

Partial symptom improvement across adolescence has been reported, particularly in social communication [53]. Yet, repetitive behaviors remain relatively intractable. This raises questions about differential neural plasticity across behavioral domains and whether certain interventions selectively target more malleable functions. Research has yet to clarify the neurobiological correlates of intervention responsiveness during adolescence.

Psychiatric comorbidities such as anxiety and ADHD significantly impact transition readiness and long-term outcomes. However, much of the comorbidity research aggregates children, adolescents, and adults, obscuring the unique patterns of comorbidity emergence and persistence during adolescence specifically. More age-stratified research is urgently needed to guide stage-appropriate interventions.

Recent advances in ASD research have deepened our understanding of atypical information processing, such as prolonged intrinsic neural timescales and their correlation with symptom severity [18]. However, these findings are often based on small samples or poorly matched control groups, limiting statistical power and generalizability. Moreover, replication studies are scarce. The reliability and clinical relevance of these biomarkers remain open questions.

Functional connectivity studies have revealed age-dependent differences in global and regional brain networks. For example, interhemispheric hypo-connectivity and regional hyper-connectivity continue to be identified, with evidence suggesting maturational differences between adolescents and adults [22,25]. Yet, inconsistencies in imaging methodology, preprocessing techniques, and region-of-interest definitions persist across studies. Task-based paradigms offer potential to resolve some of these inconsistencies, but they remain underutilized in developmental ASD research.

The role of the DMN in self-referential processing and social cognition has received increased attention. However, its developmental trajectory during adolescence—when social identity and autonomy emerge—is not well understood. Similarly, the early origins of DMN disruptions remain speculative, with few studies linking fetal or infant neural development to later adolescent outcomes [24,29].

Evidence-based intervention research continues to expand, but translation to practice remains limited, particularly in low-resource environments. While guidelines informed by multidisciplinary perspectives are being developed [48], their uptake and effectiveness in real-world settings require better evaluation. Furthermore, despite meta-analytic data on maladaptive behaviors [7], the field lacks consensus on which behaviors most impair transition outcomes and how they should be prioritized in interventions.

A particularly promising yet underexplored area is the late acquisition of daily living skills. Contrary to the longstanding belief that development plateaus in adolescence, newer studies suggest that appropriate environmental scaffolding can promote continued growth into adulthood [56]. However, mechanisms underlying this “late bloom” phenomenon remain speculative.

Finally, while the integration of genetic, neurobiological, and behavioral data is widely endorsed [1], few studies achieve this level of interdisciplinarity. Future research must prioritize larger, diverse samples and longitudinal, multimodal designs to advance precision intervention and personalized support planning [59].

### Integration of Outstanding Questions and Knowledge Gaps

Despite significant progress, several key questions remain unanswered in the study of adolescents with ASD. One pressing issue is the identification of neurobiological features that reliably predict adaptive versus maladaptive outcomes during the transition to adulthood. It is unclear which brain-based markers—such as specific connectivity patterns or developmental trajectories in white matter maturation—are most indicative of successful independence or persistent functional limitations.

Additionally, inconsistencies across studies highlight the need to critically assess how methodological variables—such as sample size, imaging modality, diagnostic thresholds, and task-based versus resting-state paradigms—contribute to the divergent findings regarding neural connectivity abnormalities in ASD. These methodological issues complicate the field’s ability to build a coherent understanding of developmental brain differences specific to adolescence.

Another important question pertains to the environmental and instructional factors that support continued acquisition of daily living skills beyond adolescence. Although emerging evidence suggests that skill growth does not universally plateau, the mechanisms by which certain adolescents continue to develop while others stagnate remain poorly understood. Identifying contextual and neurocognitive moderators of this growth is a priority [59].

The role of sociocultural and demographic variables—including gender, ethnicity, and socioeconomic status—also warrants deeper investigation. Many existing studies lack representative samples, limiting the generalizability of findings and potentially obscuring meaningful subgroups within the adolescent ASD population.

Lastly, the field still lacks consensus regarding the impact of psychiatric comorbidities, particularly ADHD and anxiety, during adolescence. Clarifying how these co-occurring conditions influence developmental trajectories, intervention responsiveness, and adult outcomes remains a critical research need [56].

Answering these questions will require longitudinal, multimodal research designs, greater methodological standardization, and stronger integration across behavioral, cognitive, neurobiological, and environmental domains.

## 9. Conclusions

ASD during adolescence presents a distinctive constellation of neurobiological, cognitive, and behavioral challenges that are both developmentally dynamic and highly heterogeneous. Throughout this review, we have identified converging evidence that highlights widespread atypicalities in brain connectivity, functional specialization, and adaptive behavior in adolescents with ASD. These include, but are not limited to, underconnectivity within and between frontoparietal and default mode networks, overconnectivity in sensorimotor and subcortical systems, delayed maturation in white matter tracts, and atypical trajectories in cognitive domains such as executive function, social processing, and emotion regulation.

What is known with reasonable confidence is that these neurobiological alterations contribute to a broad spectrum of cognitive and adaptive outcomes. Structural and functional neuroimaging consistently implicates disruptions in regions subserving higher-order cognitive control, theory of mind, and interoception. Developmental patterns, while variable, show divergence from NT norms particularly in the transition from adolescence into adulthood, where adaptive skill acquisition often plateaus and psychiatric comorbidities become more pronounced.

Yet several areas remain equivocal. Considerable variability exists in study outcomes due to methodological differences in sample characterization (e.g., IQ, sex distribution, comorbidity profiles), task design (e.g., passive viewing vs. active engagement paradigms), and neuroimaging techniques (e.g., resting-state vs. task-based fMRI). The significance of observed brain-behavior correlations is often constrained by cross-sectional designs, insufficient longitudinal data, and lack of replication. For instance, while hypo-connectivity in default mode regions is widely reported, its exact developmental onset, behavioral correlates, and plasticity remain debated.

Unknowns persist regarding the mechanisms that drive individual differences in adolescent ASD outcomes. Questions remain about which neurodevelopmental markers reliably predict positive adaptation, what role environmental modifiers play in shaping trajectories, and how interventions might be tailored to emerging adolescent needs. The extent to which neural compensation versus persistent deficits account for changes in behavior is poorly understood, and the influence of hormonal, sociocultural, and ecological transitions during adolescence on brain and behavior in ASD remains underexplored.

Given these challenges, it is clear that supporting adolescents with ASD requires both precision and breadth: precision in identifying neural and behavioral phenotypes that guide personalized interventions, and breadth in constructing systemic supports—educational, social, clinical—that can accommodate the full spectrum of needs. There is a pressing need for longitudinal, multimodal, and interdisciplinary approaches that bridge neurodevelopmental research with real-world implementation in clinical and educational settings.

Ultimately, ASD in adolescence must be understood as a lifespan issue, with adolescence representing a critical inflection point where neurobiological plasticity intersects with escalating environmental demands. Continued research that interrogates the intersection of biology, behavior, and context will be essential to refining diagnostic models, informing intervention timing and targets, and ultimately improving long-term outcomes for individuals with ASD and their families.

## Data Availability

The original contributions presented in this study are included in the article. Further inquiries can be directed to the corresponding author.

## Abbreviations

The following abbreviations are used in this manuscript:

ABIDE	Multidisciplinary Digital Publishing Institute
ACC	Anterior cingulate cortex
ADHD	Directory of open access journals
ASD	Three letter acronym
dFC	Dynamic functional connectivity
DMN	Linear dichroism
DTI	Diffusion tensor imaging
EF	Executive function
ER	Emotional regulation
FA	Fractional anisotropy
FPCN	Frontoparietal control network
fMRI	Functional magnetic resonance imaging
GSR	Global signal repression
ICA	Independent component analysis
mPFC	Medial prefrontal cortex
PCC	Posterior cingulate cortex
pSTS	Posterior superior central sulcus
RPB	Restricted repetitive behaviors
ROI	Regions of interest
RS-fMRI	Resting-state functional magnetic resonance imaging
SN	Salience network
STS	Superior temporal sulcus
TBSS	Tract-based spatial statistics
ToM	Theory of mind
TPJ	Temporoparietal junction
VBM	Voxel-based morphometry

## References

[1] Volkmar F.R., Reichow B., McPartland J.C. (2024). Adolescents and Adults with Autism Spectrum Disorders.

[2] Keun-AhCheon J.S.E. (2016). Abnormalities of inter- and intra-hemispheric functional connectivity in autism spectrum disorders: A study using the autism brain imaging data exchange database. Front. Neurosci..

[3] Picci G., Gotts S.J., Scherf K.S. (2016). A theoretical rut: Revisiting and critically evaluating the generalized under/over-connectivity hypothesis of autism. Dev. Sci..

[4] Harlalka V., Bapi R.S., Vinod P.K., Roy D. (2019). Atypical flexibility in dynamic functional connectivity quantifies the severity in autism spectrum disorder. Front. Hum. Neurosci..

[5] Xu Q., Zuo C., Liao S., Long Y., Wang Y. (2020). Abnormal development pattern of the amygdala and hippocampus from childhood to adulthood with autism. J. Clin. Neurosci..

[6] Leisman G., Melillo R., Melillo T. (2023). Prefrontal functional connectivities in autism spectrum disorders: A connectopathic disorder affecting movement, interoception, and cognition. Brain Res. Bull..

[7] Shattuck P.T., Seltzer M.M., Greenberg J.S., Orsmond G.I., Bolt D., Kring S., Lounds J., Lord C. (2006). Change in autism symptoms and maladaptive behaviors in adolescents and adults with an autism spectrum disorder. J. Autism Dev. Disord..

[8] Khundrakpam B.S., Lewis J.D., Kostopoulos P., Carbonell F., Evans A.C. (2017). Cortical thickness abnormalities in autism spectrum disorders through late childhood, adolescence, and adulthood: A large-scale MRI study. Cereb. Cortex.

[9] Arnold S., Guell X., D’Mello A., Joshi G. (2018). Disrupted cerebrocerebellar intrinsic functional connectivity in young adults with high-functioning autism spectrum disorder: A data-driven, whole-brain, high-temporal resolution functional magnetic resonance imaging study. Brain Connect..

[10] Uddin L.Q. (2020). Brain Mechanisms Supporting Flexible Cognition and Behavior in Adolescents with Autism Spectrum Disorder. Biol. Psychiatry.

[11] Ecker C. (2017). The neuroanatomy of autism spectrum disorder: An overview of structural neuroimaging findings and their translatability to the clinical setting. Autism.

[12] Ecker C., Andrews D.S., Gudbrandsen C.M. (2017). RETRACTED: Ecker et al. Association between the probability of autism spectrum disorder and normative sex-related phenotypic diversity in brain structure. JAMA Psychiatry.

[13] Wallace G.L., Dankner N., Kenworthy L., Giedd J.N., Martin A. (2010). Age-related temporal and parietal cortical thinning in autism spectrum disorders. Brain.

[14] Casanova M.F., Buxhoeveden D.P., Switala A.E., Roy E. (2002). Minicolumnar pathology in autism. Neurology.

[15] Ritvo E.R., Freeman B.J., Scheibel A.B., Duong T., Robinson H., Guthrie D., Ritvo A. (1986). Lower Purkinje cell counts in the cerebella of four autistic subjects: Initial findings of the UCLA-NSAC Autopsy Research Report. Am. J. Psychiatry.

[16] Pelphrey K.A., Shultz S., Hudac C.M., Vander Wyk B.C. (2011). Research review: Constraining heterogeneity: The social brain and its development in autism spectrum disorder: Research Review: Constraining heterogeneity. J. Child Psychol. Psychiatry.

[17] Nair A., Jolliffe M., Lograsso Y.S.S., Bearden C.E. (2020). A review of default mode network connectivity and its association with social cognition in adolescents with autism spectrum disorder and early-onset psychosis. Front. Psychiatry.

[18] Watanabe T., Rees G., Masuda N. (2019). Atypical intrinsic neural timescale in autism. Elife.

[19] O’Hearn K., Lynn A. (2023). Age differences and brain maturation provide insight into heterogeneous results in autism spectrum disorder. Front. Hum. Neurosci..

[20] Lee Y., Park B., James O., Kim S.-G., Park H. (2017). Autism spectrum disorder related functional connectivity changes in the language network in children, adolescents and adults. Front. Hum. Neurosci..

[21] Levy A., Perry A. (2011). Outcomes in adolescents and adults with autism: A review of the literature. Res. Autism Spectr. Disord..

[22] Ruan L., Chen G., Yao M., Li C., Chen X., Luo H., Ruan J., Zheng Z., Zhang D., Liang S. (2024). Brain functional gradient and structure features in adolescent and adult autism spectrum disorders. Hum. Brain Mapp..

[23] Lawrence K.E., Hernandez L.M., Bookheimer S.Y., Dapretto M. (2019). Atypical longitudinal development of functional connectivity in adolescents with autism spectrum disorder: Developmental changes in RSNs in ASD. Autism Res..

[24] Padmanabhan A., Lynch C.J., Schaer M., Menon V. (2017). The default mode network in autism. Biol. Psychiatry Cogn. Neurosci. Neuroimaging.

[25] Uddin L.Q., Supekar K., Menon V. (2013). Reconceptualizing functional brain connectivity in autism from a developmental perspective. Front. Hum. Neurosci..

[26] Haghighat H., Mirzarezaee M., Araabi B.N., Khadem A. (2021). Functional networks abnormalities in autism spectrum disorder: Age-related hypo and hyper connectivity. Brain Topogr..

[27] Morgan J.T., Barger N., Amaral D.G., Schumann C.M. (2014). Stereological study of amygdala glial populations in adolescents and adults with autism spectrum disorder. PLoS ONE.

[28] Cao M., Huang H., Peng Y., Dong Q., He Y. (2016). Toward developmental connectomics of the human brain. Front. Neuroanat..

[29] Burrows C.A., Laird A.R., Uddin L.Q. (2016). Functional connectivity of brain regions for self- and other-evaluation in children, adolescents and adults with autism. Dev. Sci..

[30] Dajani D.R. (2016). Local brain connectivity across development in autism spectrum disorder: A cross-sectional investigation. Autism Res..

[31] Redcay E., Courchesne E. (2005). When is the brain enlarged in autism? A meta-analysis of all brain size reports. Biol. Psychiatry.

[32] O’Reilly C., Lewis J.D., Elsabbagh M. (2017). Is functional brain connectivity atypical in autism? A systematic review of EEG and MEG studies. PLoS ONE.

[33] Boedhoe P.S., van Rooij D., Hoogman M., Twisk J.W., Schmaal L., Abe Y., Alonso P., Ameis S.H., Anikin A., Anticevic A. (2020). Subcortical brain volume, regional cortical thickness, and cortical surface area across disorders: Findings from the ENIGMA ADHD, ASD, and OCD working groups. Am. J. Psychiatry.

[34] Herbert M.R., Ziegler D.A., Deutsch C.K., O’brien L.M., Lange N., Bakardjiev A., Hodgson J., Adrien K.T., Steele S., Makris N. (2003). Dissociations of cerebral cortex, subcortical and cerebral white matter volumes in autistic boys. Brain.

[35] Hyde K.L., Samson F., Evans A.C., Mottron L. (2010). Neuroanatomical differences in brain areas implicated in perceptual and other core features of autism revealed by cortical thickness analysis and voxel-based morphometry. Hum. Brain Mapp..

[36] Wallace G.L., Robustelli B., Dankner N., Kenworthy L., Giedd J.N., Martin A. (2013). Increased gyrification, but comparable surface area in adolescents with autism spectrum disorders. Brain.

[37] Ecker C., Bookheimer S.Y., Murphy D.G.M. (2015). Neuroimaging in autism spectrum disorder: Brain structure and function across the lifespan. Lancet Neurol..

[38] Schumann C.M., Hamstra J., Goodlin-Jones B.L., Lotspeich L.J., Kwon H., Buonocore M.H., Lammers C.R., Reiss A.L., Amaral D.G. (2004). The amygdala is enlarged in children but not adolescents with autism; the hippocampus is enlarged at all ages. J. Neurosci..

[39] Zhang W., Groen W., Mennes M., Greven C., Buitelaar J., Rommelse N. (2017). Revisiting subcortical brain volume correlates of autism in the ABIDE dataset: Effects of age and sex. Psychol. Med..

[40] Rafiee F., Rezvani Habibabadi R., Motaghi M., Yousem D.M., Yousem I.J. (2022). Brain MRI in autism spectrum disorder: Narrative review and recent advances. J. Magn. Reson. Imaging.

[41] Waiter G.D., Williams J.H., Murray A.D., Gilchrist A., Perrett D.I., Whiten A. (2005). Structural white matter deficits in high-functioning individuals with autistic spectrum disorder: A voxel-based investigation. Neuroimage.

[42] Andrews D.S., Lee J.K., Harvey D.J., Waizbard-Bartov E., Solomon M., Rogers S.J., Nordahl C.W., Amaral D.G. (2021). A longitudinal study of white matter development in relation to changes in autism severity across early childhood. Biol. Psychiatry.

[43] Courchesne E., Mouton P.R., Calhoun M.E., Semendeferi K., Ahrens-Barbeau C., Hallet M.J., Barnes C.C., Pierce K. (2011). Neuron number and size in prefrontal cortex of children with autism. JAMA.

[44] Courchesne E., Campbell K., Solso S. (2010). Brain growth across the life span in autism: Age-specific changes in anatomical pathology. Brain Res..

[45] Betancur C. (2011). Etiological heterogeneity in autism spectrum disorders: More than 100 genetic and genomic disorders and still counting. Brain Res..

[46] Ecker C., Ronan L., Feng Y., Daly E., Murphy C., Ginestet C.E., Brammer M., Fletcher P.C., Bullmore E.T., Suckling J. (2013). Intrinsic gray-matter connectivity of the brain in adults with autism spectrum disorder. Proc. Natl. Acad. Sci. USA.

[47] Chen L., Abate M., Fredericks M., Guo Y., Tao Z., Zhang X. (2024). Age-related differences in the intrinsic connectivity of the hippocampus and ventral temporal lobe in autistic individuals. Front. Hum. Neurosci..

[48] DuBois D., Lymer E., Gibson B.E., Desarkar P., Nalder E. (2017). Assessing sensory processing dysfunction in adults and adolescents with Autism Spectrum Disorder: A scoping review. Brain Sci..

[49] Sato W., Kochiyama T., Uono S., Yoshimura S., Kubota Y., Sawada R., Sakihama M., Toichi M. (2017). Reduced gray matter volume in the social brain network in adults with autism spectrum disorder. Front. Hum. Neurosci..

[50] Guo X., Duan X., Long Z., Chen H., Wang Y., Zheng J., Zhang Y., Li R., Chen H. (2016). Decreased amygdala functional connectivity in adolescents with autism: A resting-state fMRI study. Psychiatry Res. Neuroimaging.

[51] Li L., He C., Jian T., Guo X., Xiao J., Li Y., Chen H., Kang X., Chen H., Duan X. (2021). Attenuated link between the medial prefrontal cortex and the amygdala in children with autism spectrum disorder: Evidence from effective connectivity within the “social brain”. Prog. Neuro-Psychopharmacol. Biol. Psychiatry.

[52] Hazlett H.C., Poe M.D., Gerig G., Smith R.G., Piven J. (2006). Cortical gray and white brain tissue volume in adolescents and adults with autism. Biol. Psychiatry.

[53] Mailick Seltzer M., Wyngaarden Krauss M., Shattuck P.T., Orsmond G., Swe A., Lord C. (2003). The Symptoms of Autism Spectrum Disorders in Adolescence and Adulthood. J. Autism Dev. Disord..

[54] Cheng Y., Shi J., Cheng X., Wei Y., Wang J., Jiang Z. (2025). Impact of social knowledge and skills training based on UCLA PEERS® on social communication and interaction skills of adolescents or young adults with autism: A systematic review and meta-analysis. Asian J. Psychiatr..

[55] Braden B.B., Smith C.J., Thompson A., Glaspy T.K., Wood E., Vatsa D., Abbott A.E., McGee S.C., Baxter L.C. (2017). Executive function and functional and structural brain differences in middle-age adults with autism spectrum disorder. Autism Res..

[56] Dajani D.R., Uddin L.Q. (2015). Demystifying cognitive flexibility: Implications for clinical and developmental neuroscience. Trends Neurosci..

[57] Goldman S.E., Alder M.L., Burgess H.J., Corbett B.A., Hundley R., Wofford D., Fawkes D.B., Wang L., Laudenslager M.L., Malow B.A. (2017). Characterizing sleep in adolescents and adults with autism spectrum disorders. J. Autism Dev. Disord..

[58] Woodward N.D., Giraldo-Chica M., Rogers B., Cascio C.J. (2017). Thalamocortical dysconnectivity in autism spectrum disorder: An analysis of the Autism Brain Imaging Data Exchange. Biol. Psychiatry Cogn. Neurosci. Neuroimaging.

[59] Smith L.E., Maenner M.J., Seltzer M.M. (2012). Developmental trajectories in adolescents and adults with autism: The case of daily living skills. J. Am. Acad. Child Adolesc. Psychiatry.

[60] Donovan A.P.A., Basson M.A. (2016). The neuroanatomy of autism—A developmental perspective. J. Anat..

[61] Hua X., Thompson P.M., Leow A.D., Madsen S.K., Caplan R., Alger J.R., O’NEill J., Joshi K., Smalley S.L., Toga A.W. (2013). Brain growth rate abnormalities visualized in adolescents with autism. Hum. Brain Mapp..

